# Analysis of the impact of maternal sepsis on pregnancy outcomes: a population-based retrospective study

**DOI:** 10.1186/s12884-024-06607-8

**Published:** 2024-08-01

**Authors:** Hsin-Hua Chen, Chieh-Liang Wu, Wen-Cheng Chao

**Affiliations:** 1 Division of General Medicine, Department of Internal Medicine, Taichung Veterans General Hospital, Taichung, Taiwan; 2 Big Data Center, Chung Hsing University, Taichung, Taiwan; 3 Department of post-Baccalaureate Medicine, College of Medicine, National Chung Hsing University, Taichung, Taiwan; 4 Department of Industrial Engineering and Enterprise Information, Tunghai University, Taichung, Taiwan; 5 Department of Critical Care Medicine, Taichung Veterans General Hospital, Taichung, Taiwan

**Keywords:** Maternal sepsis, Sepsis-3, Birth outcome, Perinatal outcome

## Abstract

**Background:**

To investigate the association between maternal sepsis during pregnancy and poor pregnancy outcome and to identify risk factors for poor birth outcomes and adverse perinatal events.

**Methods:**

We linked the Taiwan Birth Cohort Study (TBCS) database and the Taiwanese National Health Insurance Database (NHID) to conduct this population-based study. We analysed the data of pregnant women who met the criteria for sepsis-3 during pregnancy between 2005 and 2017 as the maternal sepsis cases and selected pregnant women without infection as the non-sepsis comparison cohort. Sepsis during pregnancy and fulfilled the sepsis-3 definition proposed in 2016. The primary outcome included low birth weight (LBW, < 2500 g) and preterm birth (< 34 weeks), and the secondary outcome was the occurrence of adverse perinatal events.

**Results:**

We enrolled 2,732 women who met the criteria for sepsis-3 during pregnancy and 196,333 non-sepsis controls. We found that the development of maternal sepsis was highly associated with unfavourable pregnancy outcomes, including LBW (adjOR 9.51, 95% CI 8.73–10.36), preterm birth < 34 weeks (adjOR 11.69, 95%CI 10.64–12.84), and the adverse perinatal events (adjOR 3.09, 95% CI 2.83–3.36). We also identified that socio-economically disadvantaged status was slightly associated with an increased risk for low birth weight and preterm birth.

**Conclusion:**

We found that the development of maternal sepsis was highly associated with LBW, preterm birth and adverse perinatal events. Our findings highlight the prolonged impact of maternal sepsis on pregnancy outcomes and indicate the need for vigilance among pregnant women with sepsis.

**Supplementary Information:**

The online version contains supplementary material available at 10.1186/s12884-024-06607-8.

## Introduction

Sepsis consists of dysregulated inflammation and life-threatening organ dysfunction. It may affect pregnant women with altered physiological and immunological responses during pregnancy, so-called maternal sepsis [1–4]. Rudd KE et al., using the Global Burden of Diseases-2017 database that included data from Austria, Brazil, Canada, Chile, Georgia, Italy, Mexico, New Zealand, Philippines and the USA, reported approximately 5.7 million pregnant women presented with maternal disorders complicated with sepsis [4]. Increasing evidence, including our study, has shown that sepsis may have a prolonged impact on patients who recovered from sepsis; however, few studies explored the association between maternal sepsis and the birth/perinatal outcome [5–8]. It is estimated that approximately 20 million infants are born with low birth weight (LBW) (< 2,500 g), and nearly 14.9 million are born preterm [9]. Notably, the aforementioned adverse birth outcome predominantly occurs in low- and middle-income countries [9, 10], and accumulating evidence, including our study, have shown that socio-economically disadvantaged status appears to be associated with incident sepsis [11–14]. Therefore, there is a crucial need to address the association between maternal sepsis and adverse birth outcomes and clarify the role of socioeconomic status. We hypothesised that sepsis during pregnancy might have a prolonged impact on the birth outcome and adverse perinatal events. In the present study, we linked two population-based claim databases, including the Taiwanese birth cohort and national health insurance databases, to investigate the association between maternal sepsis and birth and prenatal outcomes and to identify risk factors for the aforementioned adverse pregnancy outcomes.

## Materials and methods

### Data sources

The present study linked two population-based claim databases in Taiwan, including the Taiwan Birth Cohort Study (TBCS) database and the National Health Insurance Database (NHID). TBCS, initiated by the Taiwanese Health Promotion Administration, has collected birth outcomes and perinatal data in Taiwan since 2003 [15, 16]. The data regarding maternal sepsis were retrieved from the NHID in Taiwan. In brief, National Health Insurance (NHI), issued in 1997, is compulsory population-based insurance in Taiwan with comprehensive population coverage (99.96% of Taiwanese residents in 2017. The NHID has stored the original reimbursement claims data of NHI in the Health and Welfare Data Center (HWDC) [17]. Therefore, ambulatory care expenditures by visits, inpatient expenditures by admissions, expenditures for prescriptions dispensed at contracted pharmacies, details of ambulatory care orders, details of inpatient orders, details of prescriptions dispensed at contracted pharmacies, health services utilisation of medical facilities and the other needed information for this study can be used within HWDC. The medical diagnoses in NHID are based on the International Classification of Diseases, Ninth Revision, Clinical Modification (ICD-9-CM) and ICU-10-CM.

### Definition of maternal sepsis by sepsis-3

Based on previous studies, including ours, the sepsis-3 definition, which indicates organ dysfunction and the presence of infection, was used to identify patients with sepsis in the claims database [13, 18, 19]. In brief, a septic episode was defined as a diagnosis of infectious disease and at least one acute organ dysfunction. Organ dysfunction was diagnosed in accordance with the items in the sequential organ failure assessment, including dysfunction in the respiratory, cardiovascular, haematological, hepatic, renal, and central nervous systems [19, 20].

### Outcomes

The primary outcome included low birth weight (LBW, < 2500 g) and preterm birth (< 34 weeks), and the secondary outcome was the occurrence of adverse perinatal events. The adverse perinatal events were composed of fever, premature rupture of membrane, placental abruption, placenta previa, major bleeding (500 ml on normal spontaneous delivery or 1000 ml on Cesarean section), precipitate labour, malposition, umbilical cord prolapse, and fetal distress [21].

### Covariates

The potential confounders adjusted in the Cox regression model were age, socioeconomic status, comorbidities, and gestational risks, including gestational diabetes, history of preterm delivery, current smoker, cervical insufficiency and preeclampsia. Comorbidity was defined as one inpatient visit or more than three ambulatory visits with a corresponding ICD-9/10-CM code within one year before the index date. With respect to socioeconomic data, the urbanisation level of the patient’s residence was divided by the population density (people/km^2^), population ratio of elderly aged higher than 65 years, population ratio of agricultural workers, population ratio of those with educational levels of college or above, and the number of registered physicians per 100,000 subjects [13]. We also used payroll-related insured amount, divided by the median level of enrolled subjects, as a proxy measure of the patient’s socioeconomic status.

### Statistical analysis

Descriptive results were presented as means ± standard deviation for continuous variables or numbers (percentages) for categorical variables. In addition, we determined the risk of preterm birth, low birth weight and adverse perinatal events by estimating odds ratios (ORs) with 95% confidence intervals (CIs) via a multivariable logistical regression analysis after adjusting for potential confounders. All data were analysed using SAS version 9.3 (SAS Institute, Inc., Cary, NC, the USA). A p-value of < 0.05 was considered statistically significant.

## Results

### Characteristics of the study population

**Fig. 1:**
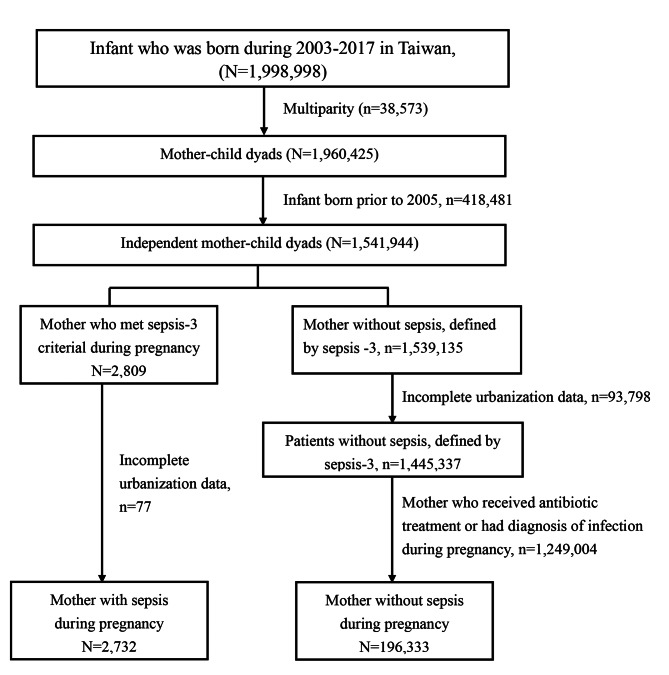
Flow chart of the subject enrollment

Figure 1 illustrates the selection of those with maternal sepsis and pregnant women without sepsis (Fig. 1). We identified 1,998,998 infants born during 2003–2017 in the TBCS and excluded multiparity. To obtain data regarding maternal comorbidity before pregnancy, we further excluded the data of infants who were born before 2005. A total of 1,541,944 independent mother-child dyads were eligible for analyses. We identified 2,809 patients with sepsis during pregnancy and excluded 77 patients without detailed urbanisation data. To avoid including those with potential infection in the non-sepsis pregnant women, we excluded those who received antibiotic treatment or were diagnosed with infection during pregnancy. A total of 196,333 were enrolled as non-sepsis controls. Table 1 summarises the characteristics of the sepsis group and the non-sepsis group. Compared with non-sepsis pregnant women, patients with maternal sepsis had higher proportions of being older or equal to 35 years (20.1% vs. 15.7%, *p* < 0.01), having a low level of urbanisation in residence (24.7% vs. 16.3%, *p* < 0.01), and having a low insured income (62% vs. 50.7%, *p* < 0.01). Those with maternal sepsis were more likely to have comorbidities, including hypertension, diabetes, hyperlipidemia, depression, hyperthyroidism, chronic liver disease and connective tissue disease, than those in the non-sepsis group.

**Table 1:** Maternal characteristics and gestational risks of the mother-child dyads with and without sepsis

	Maternal Sepsis (+)	Maternal Sepsis (-)	*p*-value
	*N* = 2,732	*N* = 196,333	
**Maternal age**	30.9 ± 5.4	30.8 ± 4.9	0.135
< 35 years	2,183 (79.9)	165,446 (84.3)	< 0.001
≥ 35 years	549 (20.1)	30,887 (15.7)	
**Gestational age (weeks)**	35.9 ± 4.3	38.3 ± 2.5	< 0.001
**Socioeconomic status**			
**Urbanisation level**			< 0.001
Urban	2,057 (75.3)	164,406 (83.7)	
Rural	675 (24.7)	31,927 (16.3)	
**Low insured income**^a^	1,694 (62)	99,582 (50.7)	< 0.001
**Sepsis timing**			
Sepsis within 1st trimester	85 (3.1)	N/A	
Sepsis after 1st trimester	2,647 (96.9)	N/A	
**Comorbidities**			
Hypertension	43 (1.6)	316 (0.2)	< 0.001
Diabetes mellitus	66 (2.4)	312 (0.2)	< 0.001
Hyperlipidemia	29 (1.1)	406 (0.2)	< 0.001
Malignancy	10 (0.4)	411 (0.2)	0.077
Depression	29 (1.1)	782 (0.4)	< 0.001
Hyperthyroidism	23 (0.8)	1,116 (0.6)	0.06
Chronic liver diseases	18 (0.7)	550 (0.3)	< 0.001
Connective tissue disease	14 (0.5)	278 (0.1)	< 0.001
**Gestational risks**			
Gestational diabetes	70 (2.6)	2,513 (1.3)	< 0.001
History of preterm delivery	26 (1.0)	144 (0.1)	< 0.001
Current smoker	16 (0.6)	180 (0.1)	< 0.001
Cervical insufficiency	22 (0.8)	87 (0.04)	< 0.001
Preeclampsia	75 (2.7)	1,107 (0.6)	< 0.001

^a^Insured income lower than median income (24,000 New Taiwan dollars)

^b^Higher than 500 ml on normal spontaneous delivery or 1000 ml on Cesarean section.

Abbreviation: N/A: Not Applicable.

Moreover, those with maternal sepsis also had more gestational risks, including gestational diabetes, history of preterm delivery, current smoker, cervical insufficiency and preeclampsia, compared with those in non-sepsis control. Concerning the pregnancy outcome, we found that those with maternal sepsis were more likely to have unfavourable birth outcomes, including LBW and preterm birth, and any one or more perinatal adverse events than pregnant women without sepsis (Table 2). Taken together, we found that those with maternal sepsis appeared to have a socio-economically disadvantaged status, more comorbidities, gestational risks, unfavourable birth outcomes, and one or more perinatal adverse events than the non-sepsis group.

**Table 2:** Birth outcomes and perinatal adverse events among enrolled mother-child dyads with and without sepsis

	Maternal Sepsis (+)	Maternal Sepsis (-)	*p*-value
	*N* = 2,732	*N* = 196,333	
**Birth outcomes**			
Birth weight (g)	2555.9 ± 869.1	3035.0 ± 523.1	< 0.001
Low birth weight (< 2500 g)	923 (33.8)	8,917 (4.5)	< 0.001
Preterm birth (< 34 weeks)	716 (26.2)	5,008 (2.6)	< 0.001
Preterm birth (< 37 weeks)	1,230 (45.0)	15,001 (7.6)	< 0.001
Stillbirth	3,273 (1.7)	47 (1.7)	0.829
**Perinatal adverse events, any**	769 (28.1)	21,036 (10.7)	< 0.001
Fever	73 (2.7)	1,303 (0.7)	< 0.001
Premature rupture of membrane	237 (8.7)	4,377 (2.2)	< 0.001
Placental abruption	69 (2.5)	712 (0.4)	< 0.001
Placenta previa	56 (2.0)	1,340 (0.7)	< 0.001
Major bleeding^b^	28 (1.0)	818 (0.4)	< 0.001
Precipitate labor	64 (2.3)	3,537 (1.8)	0.035
Malposition	250 (9.2)	7,084 (3.6)	< 0.001
Umbilical cord prolapse	9 (0.3)	307 (0.2)	0.024
Fetal distress	190 (7.0)	2,961 (1.5)	< 0.001

^a^Insured income lower than median income (24,000 New Taiwan dollars)

^b^Higher than 500 ml on normal spontaneous delivery or 1000 ml on Cesarean section.

### The association between maternal sepsis and birth outcomes, including low birth weight and preterm birth (< 34 weeks)

We then explored the association between maternal sepsis and LBW (Table 3 and Supplemental Table 1). We found that sepsis was highly associated with the LBW (adjOR 9.51, 95% CI 8.73–10.36). Notably, we also found that a low urbanisation level (adjOR 1.16, 95% CI 1.10–1.23) and a low insured income (adjOR 1.20, 95% 1.15–1.26) were slightly associated with LBW. Furthermore, we also noted that current smoking was modestly associated with LBW (adjOR 3.27, 95% CI 2.21–4.84). As expected, a history of preterm birth, cervical insufficiency and preeclampsia were strong predictors for LBW. Similar with the data in LBW, we found that maternal sepsis was also a robust predictor for preterm birth (< 34 weeks) (adjOR 11.69, 95%CI 10.64–12.84) (Table 4 and Supplemental Table 2). In line with the data in LBW, socio-economically disadvantaged status was slightly associated with preterm birth.

**Table 3:** Crude and adjusted odds ratios for the association between variables and low birth weight (< 2500 g)

	Crude OR (95% CI)	*p* value	Adjusted OR (95% CI)	*p* value
**Sepsis**	10.72 (9.88–11.64)	< 0.001	9.51 (8.73–10.36)	< 0.001
**Maternal age** ≥ **35 years**	1.59 (1.51–1.66)	< 0.001	1.50 (1.43–1.58)	< 0.001
**Urbanisation**				
Urban	Ref		Ref	
Rural	1.25 (1.19–1.32)	< 0.001	1.16 (1.10–1.23)	< 0.001
**Low insured income** ^a^	1.25 (1.20–1.30)	< 0.001	1.20 (1.15–1.26)	< 0.001
**Comorbidities**				
Hypertension	7.93 (6.31–9.97)	< 0.001	3.39 (2.56–4.49)	< 0.001
Hyperlipidemia	3.84 (2.98–4.94)	< 0.001	1.70 (1.23–2.35)	0.001
Diabetes mellitus	4.40 (3.39–5.71)	< 0.001	1.07 (0.76–1.49)	0.714
Malignancy	1.69 (1.19–2.40)	0.003	1.51 (1.05–2.17)	0.028
Depression	2.03 (1.60–2.56)	< 0.001	1.69 (1.32–2.16)	< 0.001
Hyperthyroidism	1.09 (0.84–1.41)	0.520	0.94 (0.71–1.23)	0.643
Chronic liver diseases	1.82 (1.36–2.44)	< 0.001	1.47 (1.07–2.00)	0.016
Connective tissue disease	2.72 (1.92–3.86)	< 0.001	1.88 (1.28–2.76)	0.001
**Gestational risks**				
Gestational diabetes	1.15 (0.97–1.36)	0.114	0.81 (0.67–0.97)	0.021
History of preterm delivery	13.22 (9.73–17.97)	< 0.001	8.41 (5.96–11.88)	< 0.001
Current smoker	4.80 (3.38–6.82)	< 0.001	3.27 (2.21–4.84)	< 0.001
Cervical insufficiency	16.38 (11.23–23.88)	< 0.001	11.07 (7.29–16.83)	< 0.001
Preeclampsia	14.38 (12.79–16.17)	< 0.001	12.57 (11.11–14.23)	< 0.001

^a^Insured income lower than median income (17,280 New Taiwan dollars)

**Table 4:** Crude and adjusted odds ratios for the association between variables and preterm birth (< 34 weeks)

	OR (95% CI)	*p* value	aOR (95% CI)	*p* value
**Sepsis**	13.57 (12.40–14.84)	< 0.001	11.69 (10.64–12.84)	< 0.001
**Maternal age** ≥ **35 years**	1.81 (1.70–1.93)	< 0.001	1.73 (1.63–1.85)	< 0.001
**Urbanisation**				
Urban	Ref		Ref	
Rural	1.27 (1.19–1.36)	< 0.001	1.14 (1.07–1.22)	< 0.001
**Low insured income** ^a^	1.35 (1.28–1.42)	< 0.001	1.30 (1.23–1.37)	< 0.001
**Comorbidities**				
Hypertension	8.42 (6.49–10.93)	< 0.001	2.92 (2.11–4.05)	< 0.001
Hyperlipidemia	4.32 (3.20–5.82)	< 0.001	1.64 (1.12–2.40)	0.011
Diabetes mellitus	5.92 (4.46–7.88)	< 0.001	1.35 (0.93–1.96)	0.110
Malignancy	1.78 (1.15–2.76)	0.010	1.55 (0.99–2.44)	0.057
Depression	1.53 (1.09–2.14)	0.015	1.16 (0.81–1.65)	0.413
Hyperthyroidism	1.26 (0.92–1.73)	0.143	1.07 (0.77–1.50)	0.676
Chronic liver diseases	1.96 (1.36–2.81)	< 0.001	1.51 (1.03–2.22)	0.037
Connective tissue disease	2.50 (1.59–3.93)	< 0.001	1.61 (0.98–2.64)	0.062
**Gestational risks**				
Gestational diabetes	0.98 (0.78–1.24)	0.881	0.65 (0.51–0.84)	0.001
History of preterm delivery	13.40 (9.59–18.74)	< 0.001	7.00 (4.71–10.41)	< 0.001
Current smoker	5.19 (3.43–7.84)	< 0.001	3.11 (1.94–4.99)	< 0.001
Cervical insufficiency	28.87 (19.79–42.12)	< 0.001	19.65 (12.86–30.05)	< 0.001
Preeclampsia	10.45 (9.10–12.00)	< 0.001	8.33 (7.17–9.68)	< 0.001

^a^Insured income lower than median income (17,280 New Taiwan dollars)

### The association between maternal sepsis and adverse perinatal outcomes

Regarding the perinatal outcome, we found that women with sepsis during pregnancy were modestly associated with the development of one or more adverse outcomes (adjOR 3.09, 95% CI 2.83–3.36) (Table 5 and Supplemental Table 3). In contrast with the data regarding the birth outcome, a socio-economically disadvantaged status tended to be associated with a lower risk for one or more adverse perinatal outcomes. We also noted that the comorbidity-associated risk for unfavourable birther outcomes tended to be slightly decreased in one or more adverse perinatal outcomes. Still, the gestational risks had a consistent impact on the adverse perinatal event. Therefore, maternal sepsis and gestational risks were essential for the adverse perinatal outcome.

**Table 5:** Crude and adjusted odds ratios for the association between variables and perinatal adverse event

	OR (95% CI)	*p* value	aOR (95% CI)	*p* value
**Sepsis**	3.27 (3.00–3.55)	< 0.001	3.09 (2.83–3.36)	< 0.001
**Maternal age** ≥ **35 years**	1.51 (1.46–1.56)	< 0.001	1.44 (1.39–1.50)	< 0.001
**Urbanisation**				
Urban	Ref		Ref	
Rural	0.92 (0.89–0.96)	< 0.001	0.94 (0.91–0.98)	0.004
**Low insured income** ^a^	0.86 (0.84–0.88)	< 0.001	0.87 (0.84–0.89)	< 0.001
**Comorbidities**				
Hypertension	1.77 (1.35–2.32)	< 0.001	1.04 (0.78–1.39)	0.791
Hyperlipidemia	1.54 (1.19–1.99)	0.001	0.99 (0.74–1.31)	0.934
Diabetes mellitus	2.15 (1.68–2.76)	< 0.001	1.37 (1.04–1.81)	0.025
Malignancy	1.43 (1.10–1.87)	0.009	1.28 (0.98–1.68)	0.071
Depression	1.00 (0.80–1.25)	1.000	0.93 (0.74–1.16)	0.502
Hyperthyroidism	1.28 (1.08–1.52)	0.004	1.18 (0.99–1.41)	0.054
Chronic liver diseases	1.66 (1.33–2.06)	< 0.001	1.48 (1.18–1.85)	0.001
Connective tissue disease	1.85 (1.37–2.48)	< 0.001	1.62 (1.20–2.19)	0.002
**Gestational risks**				
Gestational diabetes	2.35 (2.14–2.58)	< 0.001	2.07 (1.88–2.27)	< 0.001
History of preterm delivery	4.31 (3.14–5.92)	< 0.001	3.11 (2.23–4.34)	< 0.001
Current smoker	4.74 (3.54–6.33)	< 0.001	4.49 (3.33–6.05)	< 0.001
Cervical insufficiency	3.54 (2.35–5.32)	< 0.001	2.49 (1.62–3.84)	< 0.001
Preeclampsia	2.63 (2.30–3.01)	< 0.001	2.17 (1.90–2.50)	< 0.001

^a^Insured income lower than median income (17,280 New Taiwan dollars)

## Discussion

Maternal sepsis is an essential global health issue, particularly among pregnant women who were socio-economically disadvantaged. We linked two population-based claim databases in Taiwan to address maternal sepsis’s birth and perinatal impacts. We found that maternal sepsis was highly associated with preterm birth, LBW and adverse perinatal events. Furthermore, we identified that socioeconomic status, including income and urbanisation, was an independent risk for poor birth outcomes. Our findings demonstrate the prolonged impact of maternal sepsis, and the identified risk factors can be used in the risk stratification for pregnancy outcomes.

Few studies have explored the impact of maternal sepsis. Blauvelt et al. recently conducted a single centre study with 59 individuals who had antepartum admission for infection and 14,506 comparable control subjects and reported that antepartum sepsis correlated with a nearly 2-fold increase in odds of placental dysfunction-relevant complications, mainly hypertensive disease of pregnancy [8]. We postulate that the strengthened association between maternal sepsis and pregnancy outcome in this study may attributed by not only population-based study but also using sepsis-3, a stringent definition of sepsis restricted to septic patients with organ dysfunction. In line with our data, Blauvelt et al. also found that those with antepartum sepsis tended to have a higher proportion of preterm birth (< 34 weeks, 5.1% vs. 3.9%), although was relatively underpowered due to the small sample size. Similarly, Blauvelt et al. reported that infants of patients with antepartum sepsis tended to be small for gestational age, defined by birthweight < 10th percentile for gestational age (11.9% vs. 9.9%, *p* = 0.66). We think the high number of patients with maternal sepsis enables us to address the impact of maternal sepsis on preterm birth and LBW. We found that the majority of maternal sepsis occurred after the fir^st^ trimester, and only 3.1% (85/2,732) of sepsis developed within the first trimester of pregnancy, and this finding was in line with the study showing that the average gestational age at infection was 24.6 ± 9.0 weeks among 59 individuals with maternal sepsis at an academic referral centre in the United States [8]. Intriguingly, Blauvelt et al. found that hypertension tended to be a protective factor for poor birth outcomes in those with maternal sepsis. Our data and other data in the general population have shown that hypertension appears to be a risk factor for poor birth outcomes; therefore, more studies focusing on patients with maternal sepsis are warranted [22].

The reported prevalence of maternal sepsis varied widely with reported regions and definitions for sepsis [2, 3]. We used the sepsis-3 definition in the present study, and the sepsis-3 definition is relatively stringent and was highly associated with mortality in patients with sepsis. However, the application of sepsis-3 may underestimate some infectious patients without organ dysfunction [23]. Therefore, we used sepsis-3 in the present study given that our goal of the study is to identify critical and actionable factors of maternal sepsis. Additionally, the applied code to identify those with sepsis in claim data has been validated in previous studies, including our studies [19, 20].

Growing evidence, including our studies, has shown the previous underexplored long-term impact of sepsis [5, 7]. Sepsis during pregnancy may continue to affect the pregnancy through several post-sepsis multisystem pathophysiological alterations, so-called chronic critical illness, including muscular weakness, mental illness, and altered gastrointestinal function, including dysphagia, anorexia, diarrhoea, as well as altered microbiota [24, 25]. Our previous studies also found that culture positivity during admission may affect the long-term outcome, particularly 3–6 months after admission to the intensive care unit, in critically ill patients [26, 27]. Therefore, sepsis during pregnancy might affect the birth and perinatal outcome.

Sepsis-3 is characterised by organ dysfunction resulting from deregulated inflammation; however, few studies have addressed the potential placental dysfunction in those with maternal sepsis. Recent studies for endothelial biomarkers in sepsis have found that the activation of the angiopoietin-2/Tie-2 pathway appears to reflect the severity of organ dysfunction in sepsis [28]. Notably, both the ovine model and human studies found that the angiopoietin-2/Tie-2 pathway plays a fundament role in the development of the placenta, and an altered angiopoietin-2/Tie-2 pathway may lead to intrauterine growth restriction [29, 30]. This evidence highlights that the altered endothelial function in sepsis may affect the development of the placenta; therefore, sepsis during pregnancy may lead to dysfunction of the placenta and affect the birth and perinatal outcome.

Socioeconomic status plays a contributory role in the development of sepsis, and recent studies, including our studies, have shown the key role of socioeconomic status in patients with sepsis [9, 10]. Our recently published studies focusing on immunocompromised patients have shown that socioeconomic status plays an essential role in the development of sepsis [13, 14]. The present study further identifies that socioeconomic status is an independent risk factor for preterm delivery, LBW, and adverse perinatal events. Furthermore, several studies have suggested that the correlation between socio-economically disadvantaged status and increased infection may be attributed to the lack of insurance, increased environmental exposure to pathogens, lack of vaccination, or unhealthy behaviours, including smoking [11, 31]. In line with our finding that smoking was an independent risk factor for poor birth outcomes in pregnant women, Soneji et al. investigated the association between maternal smoking and the risk of preterm birth among 25,233,503 expectant mothers in the United States during 2011–2017 [32]. They found that smoking was associated with the risk of preterm birth [32]. Soneji et al. further demonstrated that smoking quit early in pregnancy correlated with a reduced risk of preterm birth; therefore, we think maternal smoking quit should be a modifiable factor in those with maternal sepsis. Maternal sepsis, socio-economically disadvantaged status and smoking should be attributed as crucial factors of risk stratification for pregnancy outcome.

In line with previous studies, we also found that those with depression and connective tissue disease had a slightly increased risk for adverse birth outcomes [33, 34]. As expected, a history of preterm delivery, cervical insufficiency and preeclampsia were highly associated with the development of adverse birth outcomes [35–37]. Intriguingly, gestational diabetes appeared to be a risk for preterm birth but was no longer statistically significant in multivariable analysis (Supplemental Table 4). We postulate that the adjustment of maternal hypertension and hyperlipidemia, proven risks for gestational diabetes, may lead to the discrepancy in gestational diabetes between univariable and multivariable analyses [22, 38].

Maternal sepsis is one of the substantial global health issues among pregnant women; however, the association between maternal sepsis and pregnant outcome, including birth and perinatal outcome, remains a research niche. Through linking two population-based claims, we found that maternal sepsis robustly correlated with preterm birth, LBW and perinatal adverse events. We also identified risk factors for the adverse pregnancy outcome, including socio-economically disadvantaged status. These findings should be crucial for risk stratification among pregnant women, and more studies are warranted to elucidate underlying mechanisms and explore preventive measures in pregnant women with sepsis.

The strengths of this study include the minimisation of selection bias using a population-based dataset and a large number of patients with maternal sepsis. However, there are limitations in this study. First, due to the nature of the observational study design, we could not draw a causal inference. Second, the claim database could not assess the laboratory data, microbiological findings, and body mass index. However, morbid obesity was relatively uncommon in Taiwan, and one Taiwanese population-based study using data from the Nutrition and Health Survey in Taiwan (NAHSIT) reported that the prevalence rate of morbid obesity was merely 1.4% in 2013 [39]. Third, variables regarding placental dysfunction, a key factor in linking maternal and pregnant outcomes, cannot be assessed in this study. Fourth, the number of stillbirths was small in this study, given that TBCS only enrolled those with pregnancy for longer than 20 weeks; therefore, we could not ascertain the impact of maternal sepsis on the stillbirth (Supplemental Table 5).

## Conclusions

The observational study revealed that maternal sepsis during pregnancy was highly associated with LBW, preterm birth and adverse perinatal events. Our findings highlight the prolonged impact of maternal sepsis on pregnancy outcomes and indicate the need for vigilance among pregnant women with sepsis.

### Electronic supplementary material

Below is the link to the electronic supplementary material.

Supplementary Material 1

## Data Availability

The data generated and analysed in this study are available from the corresponding author upon reasonable request.
