# Supplementary material for: Analysis of the impact of maternal sepsis on pregnancy outcomes: a population-based retrospective study
Source: BMC Pregnancy Childbirth. 2024 Aug 1;24:518. doi: 10.1186/s12884-024-06607-8 (PMC11295718; doi:10.1186/s12884-024-06607-8)
Supplement: Supplementary file 1 — Supplementary Material 1 [file 12884_2024_6607_MOESM1_ESM.pdf]

Supplemental Table 1. Maternal characteristics and gestational risks of the mother-child dyads categorized by low birth weight (<2500g)

	Low birth weight (+)<br>N=9,840	Low birth weight (-)<br>N=189,225	p value
**Sepsis**	923 (9.4)	1,809 (1.0)	<0.001
**Maternal age≥35 years**	2,219 (22.6)	29,217 (15.4)	<0.001
**Urbanisation**			<0.001
Urban	7,920 (80.5)	158,543 (83.8)	
Rural	1,920 (19.5)	30,682 (16.2)	
**Low insured income**[a]	5,533 (56.2)	95,743 (50.6)	<0.001
**Comorbidities**			
Hypertension	104 (1.1)	255 (0.1)	<0.001
Hyperlipidemia	72 (0.7)	363 (0.2)	<0.001
Diabetes mellitus	70 (0.7)	308 (0.2)	<0.001
Malignancy	34 (0.3)	387 (0.2)	0.003
Depression	77 (0.8)	734 (0.4)	<0.001
Hyperthyroidism	61 (0.6)	1,078 (0.6)	0.520
Chronic liver diseases	49 (0.5)	519 (0.3)	<0.001
Connective tissue disease	36 (0.4)	256 (0.1)	<0.001
**Gestational risks**			
Gestational diabetes	145 (1.5)	2,438 (1.3)	0.114
History of preterm delivery	69 (0.7)	101 (0.1)	<0.001
Current smoker	39 (0.4)	157 (0.1)	<0.001
Cervical insufficiency	50 (0.5)	59 (0.03)	<0.001
Preeclampsia	492 (5.0)	690 (0.4)	<0.001

Data were presented as number (percentage)

[a]Insured income lower than median income (17,280 New Taiwan dollars)

Supplemental Table 2. Maternal characteristics and gestational risks of the mother-child dyads categorized by preterm birth (<34 weeks)

	Pre-term birth (+) N=5,724	Pre-term birth (-) N=193,341	p value
**Sepsis**	716 (12.5)	2,016 (1.0)	<0.001
**Maternal age≥35 years**	1,431 (25.0)	30,005 (15.5)	<0.001
**Urbanisation**			<0.001
Urban	4,588 (80.2)	161,875 (83.7)	
Rural	1,136 (19.8)	31,466 (16.3)	
**Low insured income**[a]	3,330 (58.2)	97,946 (50.7)	<0.001
**Comorbidities**			
Hypertension	71 (1.2)	288 (0.1)	<0.001
Hyperlipidemia	49 (0.9)	386 (0.2)	<0.001
Diabetes mellitus	56 (1.0)	322 (0.2)	<0.001
Malignancy	21 (0.4)	400 (0.2)	0.009
Depression	35 (0.6)	776 (0.4)	0.014
Hyperthyroidism	41 (0.7)	1,098 (0.6)	0.142
Chronic liver diseases	31 (0.5)	537 (0.3)	<0.001
Connective tissue disease	20 (0.3)	272 (0.1)	<0.001
**Gestational risks**			
Gestational diabetes	73 (1.3)	2,510 (1.3)	0.880
History of preterm delivery	48 (0.8)	122 (0.1)	<0.001
Current smoker	26 (0.5)	170 (0.1)	<0.001
Cervical insufficiency	50 (0.9)	59 (0.03)	<0.001
Preeclampsia	270 (4.7)	912 (0.5)	<0.001

Data were presented as number (percentage)

[a]Insured income lower than median income (17,280 New Taiwan dollars)

Supplemental Table 3. Maternal characteristics and gestational risks of the mother-child dyads categorized by the presence of perinatal adverse event

	**Perinatal adverse event (+)** N=21,805	**Perinatal adverse event (-)** N=177,260	**p value**
**Sepsis**	769 (3.5)	1,963 (1.1)	<0.001
**Maternal age≥35 years**	4,630 (21.2)	26,806 (15.1)	<0.001
**Urbanisation**			<0.001
Urban	18,437 (84.6)	148,026 (83.5)	
Rural	3,368 (15.4)	29,234 (16.5)	
**Low insured income**[a]	10,388 (47.6)	90,888 (51.3)	<0.001
**Comorbidities**			
Hypertension	64 (0.3)	295 (0.2)	<0.001
Hyperlipidemia	69 (0.3)	366 (0.2)	0.001
Diabetes mellitus	79 (0.4)	299 (0.2)	<0.001
Malignancy	63 (0.3)	358 (0.2)	0.008
Depression	89 (0.4)	722 (0.4)	0.985
Hyperthyroidism	155 (0.7)	984 (0.6)	0.004
Chronic liver diseases	96 (0.4)	472 (0.3)	<0.001
Connective tissue disease	54 (0.2)	238 (0.1)	<0.001
**Gestational risks**			
Gestational diabetes	573 (2.6)	2,010 (1.1)	<0.001
History of preterm delivery	58 (0.3)	112 (0.1)	<0.001
Current smoker	72 (0.3)	124 (0.1)	<0.001
Cervical insufficiency	33 (0.2)	76 (0.04)	<0.001
Preeclampsia	287 (1.3)	895 (0.5)	<0.001

Data were presented as number (percentage)

[a]Insured income lower than median income (17,280 New Taiwan dollars)

Supplemental Table 4. Crude and adjusted odds ratios for the association between variables and preterm birth (<37 weeks)

	OR (95% CI)	p value	aOR (95% CI)	p value
**Sepsis**	9.90 (9.16–10.69)	<0.001	8.81 (8.14–9.54)	<0.001
**Maternal age≥35 years**	1.54 (1.49–1.61)	<0.001	1.49 (1.43–1.55)	<0.001
**Urbanisation**				
Urban	Ref		Ref	
Rural	1.24 (1.19–1.29)	<0.001	1.16 (1.11–1.21)	<0.001
**Low insured income**[a]	1.27 (1.23–1.32)	<0.001	1.24 (1.20–1.28)	<0.001
**Comorbidities**				
Hypertension	7.09 (5.73–8.77)	<0.001	3.31 (2.57–4.25)	<0.001
Hyperlipidemia	3.47 (2.78–4.34)	<0.001	1.46 (1.10–1.94)	0.008
Diabetes mellitus	5.54 (4.47–6.87)	<0.001	2.00 (1.52–2.63)	<0.001
Malignancy	1.66 (1.25–2.21)	0.001	1.51 (1.12–2.02)	0.007
Depression	1.89 (1.55–2.30)	<0.001	1.59 (1.30–1.96)	<0.001
Hyperthyroidism	1.08 (0.87–1.32)	0.493	0.96 (0.77–1.19)	0.684
Chronic liver diseases	1.54 (1.19–1.98)	0.001	1.23 (0.94–1.61)	0.129
Connective tissue disease	2.22 (1.63–3.03)	<0.001	1.66 (1.19–2.33)	0.003
**Gestational risks**				
Gestational diabetes	1.27 (1.12–1.45)	<0.001	0.99 (0.86–1.13)	0.832
History of preterm delivery	11.32 (8.38–15.29)	<0.001	7.59 (5.46–10.54)	<0.001
Current smoker	5.23 (3.87–7.07)	<0.001	3.89 (2.81–5.39)	<0.001
Cervical insufficiency	11.09 (7.62–16.15)	<0.001	7.54 (5.00–11.38)	<0.001
Preeclampsia	10.69 (9.52–11.99)	<0.001	9.22 (8.17–10.39)	<0.001

[a]Insured income lower than median income (17,280 New Taiwan dollars)

Supplemental Table 5. Crude and adjusted odds ratios for the association between variables and stillbirth

	OR(95% CI)	p value	aOR(95% CI)	p value
**Sepsis**	1.03 (0.77–1.38)	0.826	0.82 (0.61–1.11)	0.201
**Maternal age≥35 years**	1.83 (1.69–1.98)	<0.001	1.84 (1.70–2.00)	<0.001
**Urbanisation**				
Urban	Ref		Ref	
Rural	1.19 (1.09–1.30)	<0.001	1.14 (1.04–1.25)	0.004
**Low insured income**[a]	1.30 (1.21–1.39)	<0.001	1.30 (1.21–1.40)	<0.001
**Comorbidities**				
Hypertension	4.44 (2.95–6.68)	<0.001	2.53 (1.62–3.96)	<0.001
Hyperlipidemia	3.31 (2.17–5.04)	<0.001	1.81 (1.11–2.96)	0.017
Diabetes mellitus	3.84 (2.52–5.86)	<0.001	2.06 (1.26–3.36)	0.004
Malignancy	2.03 (1.19–3.47)	0.009	1.87 (1.09–3.19)	0.023
Depression	1.88 (1.26–2.81)	0.002	1.62 (1.08–2.42)	0.019
Hyperthyroidism	0.95 (0.59–1.51)	0.822	0.89 (0.55–1.41)	0.609
Chronic liver diseases	1.50 (0.88–2.55)	0.136	1.26 (0.73–2.15)	0.410
Connective tissue disease	1.24 (0.55–2.78)	0.606	1.15 (0.51–2.58)	0.742
**Gestational risks**				
Gestational diabetes	0.34 (0.21–0.57)	<0.001	0.29 (0.18–0.49)	<0.001
History of preterm delivery	1.79 (0.73–4.36)	0.201	1.05 (0.41–2.70)	0.921
Current smoker	2.84 (1.46–5.55)	0.002	2.41 (1.23–4.74)	0.011
Cervical insufficiency	12.51 (7.62–20.55)	<0.001	11.66 (7.01–19.42)	<0.001
Preeclampsia	1.70 (1.20–2.41)	0.003	1.41 (0.99–2.01)	0.059

[a]Insured income lower than median income (17,280 New Taiwan dollars)
